# Decoding the genetic and functional diversity of the DSF quorum-sensing system in *Stenotrophomonas maltophilia*

**DOI:** 10.3389/fmicb.2015.00761

**Published:** 2015-07-28

**Authors:** Pol Huedo, Daniel Yero, Sònia Martinez-Servat, Àngels Ruyra, Nerea Roher, Xavier Daura, Isidre Gibert

**Affiliations:** ^1^Institut de Biotecnologia i de Biomedicina (IBB), Universitat Autònoma de Barcelona (UAB)Barcelona, Spain; ^2^Departament de Genètica i de Microbiologia, Universitat Autònoma de BarcelonaBarcelona, Spain; ^3^Catalan Institution for Research and Advanced StudiesBarcelona, Spain

**Keywords:** *rpf* cluster, virulence in zebrafish, fatty acids, DSF bioassay, social cheating, bacterial cross-talk

## Abstract

*Stenotrophomonas maltophilia* uses the Diffusible Signal Factor (DSF) quorum sensing (QS) system to mediate intra- and inter-specific signaling and regulate virulence-related processes. The components of this system are encoded by the *rpf* cluster, with genes *rpfF* and *rpfC* encoding for the DSF synthase RpfF and sensor RpfC, respectively. Recently, we have shown that there exist two variants of the *rpf* cluster (*rpf*-1 and *rpf*-2), distinguishing two groups of *S. maltophilia* strains. Surprisingly, only *rpf*-1 strains produce detectable DSF, correlating with their ability to control biofilm formation, swarming motility and virulence. The evolutive advantage of acquiring two different *rpf* clusters, the phylogenetic time point and mechanism of this acquisition and the conditions that activate DSF production in *rpf*-2 strains, are however not known. Examination of this cluster in various species suggests that its variability originated most probably by genetic exchange between rhizosphere bacteria. We propose that *rpf*-2 variant strains make use of a strategy recently termed as “social cheating.” Analysis of cellular and extracellular fatty acids (FAs) of strains E77 (*rpf*-1) and M30 (*rpf*-2) suggests that their RpfFs have also a thioesterase activity that facilitates the release of unspecific FAs to the medium in addition to DSF. Production of DSF in *rpf*-1 strains appears in fact to be modulated by some of these extracellular FAs in addition to other factors such as temperature and nutrients, while in *rpf-2* strains DSF biosynthesis is derepressed only upon detection of DSF itself, suggesting that they require cohabitation with DSF-producer bacteria to activate their DSF regulatory machinery. Finally, we show that the mixed *rpf*-1/*rpf*-2 population presents synergism in DSF production and virulence capacity in an *in vivo* infection model. Recovery and quantification of DSF from co-infected animals correlates with the observed mortality rate.

## Introduction

Quorum Sensing (QS) refers to bacterial communication processes that allow populations to synchronize gene expression when reaching a critical cellular density. These communication systems rely on the production and detection of signal molecules by which bacteria can coordinate a global response and promptly adapt to environmental fluctuations. The QS system described for the nosocomial pathogen *Stenotrophomonas maltophilia* is based on the fatty acid (FA) signal DSF (*cis*-11-methyl-2-dodecenoic acid) (Fouhy et al., 2007; Huang and Lee Wong, 2007; Huedo et al., 2014b). This cell-cell communication system was described for the first time in the phytopathogen *Xanthomonas campestris* pv. *campestris* (*Xcc*) as a novel regulation mechanism of virulence-factor synthesis (Barber et al., 1997). Its components are arrayed in the *rpf* (Regulation of Pathogenicity Factors) cluster, which includes the genes *rpfB* (fatty acyl-CoA ligase), *rpfF* (DSF synthase), *rpfC* (hybrid histidine-kinase receptor and effector), and *rpfG* (cytoplasmic regulator element) (Barber et al., 1997; Slater et al., 2000; Cheng et al., 2010).

In the past few years it has been shown that DSF-family signals are widespread (He and Zhang, 2008). This QS system has been well studied in *Xanthomonas* spp. (Barber et al., 1997; Slater et al., 2000; Dow et al., 2003; He et al., 2006; Ryan et al., 2006; He and Zhang, 2008; Cheng et al., 2010), *Xylella fastidiosa* (Chatterjee et al., 2008; Beaulieu et al., 2013; Ionescu et al., 2013) and *Burkholderia* sp. (Deng et al., 2009, 2010, 2013; McCarthy et al., 2010). In *S. maltophilia*, it has been reported that DSF-QS regulates bacterial motility (Fouhy et al., 2007; Huang and Lee Wong, 2007; Huedo et al., 2014b), biofilm development (Fouhy et al., 2007; Huedo et al., 2014b), antibiotic resistance (Fouhy et al., 2007), and virulence (Fouhy et al., 2007; Huedo et al., 2014b). Nonetheless, little is known about the mechanisms participating in the synthesis and perception of DSF molecules by *S. maltophilia*.

In a recent work we have reported that, although the DSF-QS system is found in all *S. maltophilia* strains analyzed, two populations can be clearly distinguished based on the *rpf* cluster they harbor—which we have named *rpf*-1 and *rpf*-2 (Huedo et al., 2014b)—, entangling the understanding of the QS system in this bacterium. We have shown that the two *rpf* clusters differ mainly in the region encoding the N-terminus of both the synthase RpfF and the sensor RpfC. Moreover, there exists a full association between the RpfF and RpfC variants, so that in the studied population we always observed the same RpfF/RpfC combination (called RpfF-1/RpfC-1 for the *rpf*-1 cluster and RpfF-2/RpfC-2 for the *rpf*-2 cluster). Strains with the RpfF-1/RpfC-1 combination are more commonly isolated, representing nearly 60% of the tested population (Huedo et al., 2014b). Interestingly, only those strains harboring the *rpf*-1 variant produce detectable DSF levels under the assayed conditions. The conditions that may lead to DSF production and perception in the *rpf*-2 variant group, which also includes clinically relevant strains, are not yet known.

In *Xcc*, RpfF activity is regulated by RpfC, whose REC domain (CheY-like receiver domain) interacts with the RpfF substrate-binding domain blocking DSF production (Cheng et al., 2010). Therefore, dissociation of the RpfF-RpfC complex is necessary to liberate free-active RpfF and produce DSF. The high homologies of the corresponding components in *S. maltophilia*, as well as our previous results (Huedo et al., 2014b), strongly suggest that a similar mechanism regulates DSF production in *S. maltophilia*.

In this study, we have investigated further the environmental conditions that may modulate DSF production in model strains belonging to each *rpf* variant group, in particular, temperature, medium composition and presence of other FAs. These factors, notably the presence of 13-methyl-tetradecanoic acid (*iso*-15:0), affect DSF synthesis only in the *rpf*-1 variant strain. We have also characterized cellular and extracellular FAs in wild-type and Δ*rpfF* mutant strains of each variant. The dependence of some of these FAs on the expression of the *rpfF* gene suggests that both RpfF variants have an unspecific thioesterase activity, as previously found for the *Burkholderia cenocepacia* homolog, cleaving a variety of acyl-ACP (acyl-acyl carrier protein) substrates and releasing FAs that are then secreted to the medium. Finally, we also show that in *S. maltophilia* DSF is produced in a positive-feedback manner and that the two *rpf*-variant groups act synergistically by enhancing the DSF production and virulence potential of a mixed *rpf*-variant population during *in vivo* infection.

## Materials and methods

### *rpf* cluster comparison and phylogenetic tests

Sequences of the *rpf* cluster and corresponding genes from selected Xanthomonadales strains were retrieved from their respective NCBI genome sequences [http://www.ncbi.nlm.nih.gov/genome/]. The recently described genome sequence of *S. maltophilia* M30 (Huedo et al., 2014a) was used as a reference for the *rpf*-2 variant. To manually confirm some annotations, orthology relations were defined as reciprocal best matches by blastn and blastx. Concatenated nucleotide sequences for the *rpfF* and *rpfC* genes were aligned using ClustalW as implemented in MEGA 6 (Tamura et al., 2013) with default parameters. *rpfH* was also included in the analysis, if present. Neighbour-joining trees were generated and displayed using MEGA 6. The GARD program (Kosakovsky Pond et al., 2006) was used to search for putative recombination breakpoints in sequence alignments. Detailed information about the sequences analyzed in recombination tests is found in Supplementary Material (File S1).

### Bacterial strains and growth conditions

All bacterial strains used in this study are listed in Table 1. *S. maltophilia* strains E77 (*rpf*-1) and M30 (*rpf*-2) (Ferrer-Navarro et al., 2013) and their respective Δ*rpfF* mutants (Huedo et al., 2014b) were taken as model strains to investigate the molecular mechanisms underlying DSF production and perception in each *rpf* cluster variant. *X. campestris* pv. *campestris* (*Xcc*) strain 8523 pL6engGUS was used as a reporter strain to detect DSF activity and was provided by the authors (Slater et al., 2000). *S. maltophilia* strains were routinely grown at 30°C in Luria-Bertani (LB) medium on a rotary shaker. When needed, Δ*rpfF* mutants were grown in LB supplemented with erythromycin (Erm) at 500 μg/mL. The *Xcc* reporter strain was routinely grown at 28°C in NYG medium (0.5% peptone. 0.3% yeast extract and 2% glycerol) supplemented with Tc 10 μg/mL. To investigate the role of diverse FAs in DSF production by *S*. *maltophilia*, strains E77 and M30 were grown in 150 mL LB liquid cultures to an optical density of 0.1 at 600 nm. Subsequently, cultures were supplemented with diverse FAs, including lauric acid (12:0), myristic acid (14:0), 13-methyl-tetradecanoic acid (*iso*-15:0), palmitic acid (16:0), and stearic acid (18:0) (Sigma-Aldrich), at 5 μM concentration and incubated at 30°C for 24 h. Extraction and quantification of DSF molecules was done as described below.

**Table 1:** **Strains used in this study**.

**Strains**	**Relevant characteristics**	**References**
***S. maltophilia***		
E77	Wild type, *rpf*-1 cluster variant	Ferrer-Navarro et al., 2013
M30	Wild type, *rpf*-2 cluster variant	Ferrer-Navarro et al., 2013
E77 Δ*rpfF*	E77 Δ*rpfF* mutant, *Erm*^*r*^	Huedo et al., 2014b
M30 Δ*rpfF*	M30 Δ*rpfF* mutant, *Erm*^*r*^	Huedo et al., 2014b
E77 Δ*rpfF* p*rpfF*E77	E77 Δ*rpfF* mutant harboring pBBR1MCS1-Cm-*rpfF* from E77, *Cm*^*r*^ *Erm*^*r*^	Huedo et al., 2014b
M30 Δ*rpfF* p*rpfF*M30	M30 Δ*rpfF* mutant harboring pBBR1MCS1-Cm-*rpfF* from M30, *Cm*^*r*^ *Erm*^*r*^	Huedo et al., 2014b
***X. campestris* pv**. ***campestris***		
*Xcc* 8523	Δ*rpfF* mutant. DSF reporter strain. carring plasmid pL6engGUS, *Tc*^*r*^*. Kan*^*r*^	Slater et al., 2000

### Colony-based DSF bioassay

DSF determination was performed using strain *Xcc* 8523 pL6engGUS (DSF-reporter strain) as previously described (Slater et al., 2000), with few modifications. Briefly, the DSF-reporter strain was grown in 10 mL of NYG medium supplemented with Tc (10 μg/mL) to an optical density of 0.7 at 600 nm. Cells were harvested and reconstituted with 1 mL of fresh NYG and added to 100 mL of temperate NYG medium, giving a final OD600nm of 0.07, containing 1% of BD Difco Agar Noble (NYGA) and supplemented with 80 μg/mL X-Glu (5-Bromo-4-chloro-3-indolyl β-D-glucopyranoside) (Sigma) and then plated into petri plates upon solidification. Candidate strains were pin inoculated onto NYGA plates containing 80 μg/mL X-Glu and the DSF-reporter strain and incubated for 24 h at 28°C. Presence of a blue halo around the colony indicated DSF activity.

### DSF extraction from culture supernatants

For liquid and supernatant-based DSF bioassay, bacterial cultures were grown in 250 mL of LB for 48 h at 30°C (OD600 nm about 4). Supernatants were extracted with the ethyl acetate method (Barber et al., 1997) and residues were dissolved in 200 μL of 30% methanol.

### Supernatant-based DSF bioassay

Three microliters of each sample were deposited into hand-generated wells (3 mm in diameter) in 5.5 cm petri plates containing NYGA supplemented with 80 μg/mL of X-Glu and seeded with the DSF-reporter strain previously prepared to a final optical density of 0.07 at 600 nm. Plates were incubated for 24 h at 28°C. DSF activity was determined by the presence of a blue halo around the well.

### Microtitter DSF bioassay

Two-hundred microliters of a DSF reporter solution consisting of a suspension of the *Xcc* 8523 pL6engGUS strain (adjusted to an optical density of 0.07 at 600 nm) in NYG medium and supplemented with X-Glu (80 μg/mL), were deposited into wells of sterile 96-well flat-bottomed microtitter plates (BrandTech 781662). As a standard curve, synthetic DSF was added in duplicate to separate wells at different increasing concentrations (from 0.05 to 1.5 mM) and incubated at 28°C for 24 h. After incubation, the presence of DSF molecules turned the reaction color to blue with intensity (620 nm) proportional to the initial concentration of DSF (see Supplementary Material, Figure S1). Test samples were dissolved in methanol and then tested in this bioassay. Serial dilutions of test samples were added into wells and after incubation the absorbance was read at 620 nm, as done for the calibration curve. Finally, DSF quantification was done using the standard calibration curve.

### Determination of DSF production in mixed and DSF-supplemented *S. maltophilia* liquid cultures

Overnight cultures of *S. maltophilia* E77 and M30 grown in LB at 30°C were combined in a single shake flask containing 150 mL of fresh LB medium to a final OD of 0.1 each at 600 nm, to obtain a mixed initial culture (E77:M30, 1:1). The mixed culture was incubated at 30°C for 24 h and prior to DSF extraction the CFUs of each strain were evaluated by serial dilution and colony counting (note that the colony morphology of strains E77 and M30 can be distinguished). Direct specific PCR on colonies were done if necessary to corroborate strain identity. At this time point both strains reached similar number of CFU/mL. Then, culture supernatants were extracted as described above and DSF quantification was done by microtitter DSF bioassay. To validate the synergic role of synthetic DSF in the production of DSF in strain M30, initial 150 mL LB cultures of M30 (optical density of 0.1 at 600 nm) were supplemented with 0.05 μM of synthetic DSF and incubated at 30°C for 24 h. Culture supernatants were extracted as described above and DSF quantification was done by microtitter DSF bioassay. The same volume of the media supplemented with 0.05 μM DSF was extracted and quantified by microtitter DSF bioassay as control.

### Analysis of fatty acids

Analysis of total cellular FAs was carried out by the Identification Service of the DMSZ, Braunschweig, Germany, as follows. FA methyl esters were obtained from 40 mg cells scraped from Petri dishes by saponification, methylation and extraction using minor modifications of the method of Miller (Miller, 1982) and Kuykendall (Kuykendall et al., 1988). The FA methyl ester mixtures were separated using Sherlock Microbial Identification System (MIS) (MIDI, Microbial ID, Newark, DE 19711 U.S.A.), which consists of an Agilent 6890N gas chromatograph equipped with a 5% phenyl-methyl silicone capillary column (0.2 mm × 25 m), a flame ionization detector, an Agilent 7683A automatic sampler and a computer with the MIDI data base. Peaks were automatically integrated and FA names and percentages calculated by the MIS Standard Software (Microbial ID). The gas chromatographic parameters were as follows: carrier gas, ultra-high-purity hydrogen: column head pressure, 60 kPa: injection volume, 2 μL: column split ratio, 100:1: septum purge, 5 mL/min: column temperature. 170 to 270°C at 5°C/min: injection port temperature, 240°C: and detector temperature, 300°C. To analyze FAs present in the medium, bacterial cultures were grown in 2 L of LB for 48 h at 30°C with vigorous shaking (250 rpm). Cultures were centrifuged, and supernatants were extracted by the ethyl acetate method. Dry residues were dissolved in 3 mL of dichloromethane and esterified to generate FA methyl esters. Esterified extracellular FAs were identified by gas chromatography (GC) (Agilent Technologies 6890) with an Agilent 19091S-433 column coupled to a mass spectrometer (MS) detector (Hewlett-Packard 5973).

### Determination of virulence in the adult zebrafish infection model

Adult (9–12 months) wild-type zebrafish (*Danio rerio*) were kept in a 12 h light:12 h dark cycle at 28°C and fed twice daily with dry food. All fish used in infection experiments were transferred to an isolated system and acclimated for 3 days before infection. Adult zebrafish (*n* = 16 per condition) were infected intraperitoneally (Kinkel et al., 2010) with 20 μL of a bacterial suspension corresponding approximately to 50% of lethal dose (LD50). The LD50 for M30 and E77 was previously estimated in zebrafish by injecting 20 μL of bacterial suspensions at concentrations ranging from 10^8^ to 10^10^ CFU/mL and proved to be very similar (1–5 × 10^8^ CFU per animal). Four conditions were tested, including animals injected with a single dose corresponding to the LD50 of an axenic inoculum of E77, an axenic inoculum of M30, a mixed inoculum of E77 and M30 (1:1) and a mixed inoculum of mutants Δ*rpfF*-1:Δ*rpfF-2* (1:1). Each inoculum consisted in 20 μL of a bacterial suspension previously adjusted to approximately 6–7 × 10^9^ CFU/mL in sterile PBS. Mixed inocula were prepared by mixing equal amounts of adjusted bacterial suspensions. *S. maltophilia* strains were previously grown at 28°C in Columbia blood agar plates (BioMérieux) for 20 h and collected directly from the plates with sterile phosphate buffered saline (PBS). Two control groups were injected with PBS and showed no mortality. Fishes were observed daily for signs of disease and mortality.

### DSF detection and quantification from infected animal tissues

Two fishes from each experimental group (E77; M30; E77:M30 and Δ*rpfF*-1:Δ*rpfF*-2, plus two PBS-injected animals) were randomly chosen and sacrificed by an overdose of anesthetic solution (MS-222, 220 ppm) 48 h post infection (hpi). Since in the mixed wild-type inoculum all fishes died within the first 48 h, this was chosen as the time point for evaluating DSF production inside the infected animals. Two dead fishes from each group were sacrificed and introduced into their corresponding falcon tube containing 15 mL of PBS and homogenized using a Politron Homogenizer (MARK). The homogenized solution was extracted twice with the same volume of ethyl acetate and subsequently dried. Dry residues were dissolved in 100 μL of 30% methanol and DSF activity was detected and quantified by microtitter and supernatant-based DSF bioassays.

### Statistical analysis

Statistical analyses were performed using the GraphPad Prism software version 5.00. Comparison of phenotypic data was performed by One-Way analysis of variance (ANOVA) with Bonferroni's multiple comparison post-test or unpaired *t*-test with Welch correction for unequal variances. Survival curves of zebrafish infection experiments were analyzed using the Kaplan-Meier method and differences were evaluated using the log-rank test.

### Ethics statements

Zebrafish were handled in compliance with Directive 2010/63/EU of the European Parliament and of the Council on the protection of animals used for scientific purposes and with Decree 214/1997 of the Government of Catalonia, which regulates the use of animals for experimental and other scientific purposes. Experimental protocols have been reviewed and approved by the Animal and Human Experimentation Ethics Committee (CEEAH) of Universitat Autònoma de Barcelona (UAB), Spain (ref #CEEAH-1968).

## Results

### The two *rpf* cluster variants in *S. maltophilia* may have originated by horizontal exchange

**Figure 1:**
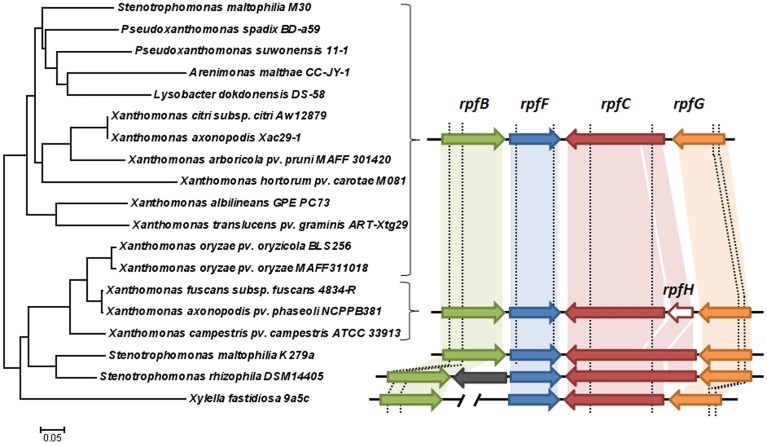
**Left:** neighbor-joining tree illustrating the relationships across Xanthomonadales strains based on concatenated coding sequences (nucleotide alignment) of the *rpfF* and *rpfC* genes. **Right**: comparison of the cluster of genes involved in DSF synthesis and perception among the analyzed strains. Arrows with the same colors indicate orthologies. The gray arrow represents a gene with unknown function unique to *S. rhizophila* DSM14405 (Alavi et al., 2014). Note that the 5′ portions of the *rpfC* coding sequence in K279a and DSM14405 strains are similar to the *rpfH* gene (conserved regions are paired with a shaded block of the same color). In *X. fastidiosa, rpfB* is located elsewhere in the chromosome. The vertical dashed lines represent recombination breakpoint sites predicted with GARD (Kosakovsky Pond et al., 2006).

To infer the origin of the two *rpf* cluster variants in *S. maltophilia* the diversity of this cluster among Xanthomonadales species was studied. In addition to *S. maltophilia* strains K279a (*rpf*-1) and M30 (*rpf*-2), 16 different species with available genome information were included in the analysis (Figure 1). In all the species analyzed, *rpfC* and *rpfF* are part of two contiguous but convergent operons, both involved in DSF synthesis and perception. Among the genes in the two *rpf* clusters, *rpfC* and *rpfF* are the most variable and were therefore selected for phylogenetic analysis. A phylogenetic tree based on the *rpfC* and *rpfF* concatenated sequences (Figure 1) suggests that the origin of the two *rpf* variants in *S. maltophilia* may be explained by horizontal gene transfer. In this tree, *S. maltophilia* K279a clusters together with *Stenotrophomonas rhizophila*, while M30 occupies a distinct, more distant branch, together with *Pseudoxanthomonas* spp., *Arenimonas* spp. and *Lysobacter* spp. strains. The nucleotide sequence of the *rpfF* gene of strain M30 (*rpf*-2 variant) is more similar to that of *Pseudoxanthomonas* spp. (80% identity) than to that of the *S. maltophilia rpf*-1 variant strain K279a (71% identity). In addition, the *rpfC* gene of *S. maltophilia* K279a and *S. rhizophila* are similar in that both encode a protein with a sensor input domain with ten transmembrane regions (TMR). This input domain is encoded by a repetitive region at the 5′ end of the gen, similar to the *rpfH* of *Xcc*. On the other hand, the *rpfC* gene of *S. maltophilia* M30 encodes a shorter protein with only five TMRs (Huedo et al., 2014b), as in most Xanthomonadales. The tree topology also suggests that a similar genetic event could have acted on the *rpf* cluster in the *Xanthomonas axonopodis* clade, as it has been already suggested (Lu et al., 2008). The presence of putative recombination breakpoints within *rpf* genes was confirmed using a genetic algorithm for recombination detection (see Figure 1 and File S1).

### Characterization of cellular and extracellular fatty acids for the two *rpf* variant strains

Analysis of total cellular FAs revealed that the *rpf*-1 and *rpf*-2 variant strains display a very heterogeneous but similar FA profile, with near 20 different FAs (Table 2). In both cases, the most abundant FA was found to be 13-methyl-tetradecanoic acid (*iso*-15:0). The presence of *iso*-15:0 as a major FA is characteristic of the genus *Stenotrophomonas* (Kim et al., 2010). Such high representation (ca. 33%) indicates that this branched FA is part of cell-membrane phospholipids (resulting from FA methyl ester extraction). Total cellular FAs were also evaluated for the Δ*rpfF*-1 and Δ*rpfF*-2 mutants and no differences were observed compared to the FA profile of their respective parental strain (Table S1).

**Table 2:** **Total cellular fatty acids in *S. maltophilia* strains E77 (*rpf*-1) and M30 (*rpf*-2)**.

**E77 (*rpf*-1)**		**M30 (*rpf*-2)**	
**Fatty acid**	**Percentage**	**Fatty acid**	**Percentage**
*iso*-15:0	32.3	*iso*-15:0	33.29
*iso*-15:0 2OH or 16:1 w7c	12.6	*iso*-15:0 2OH or 16:1 w7c	11.94
*anteiso*-15:0	11.9	*anteiso*-15:0	10.35
16:0	5.95	16:0	9.23
*iso*-17:1 w9c	4.22	*iso*-11:0	4.22
16:1 w9c	3.86	*iso*-17:1 w9c	3.95
14:0	3.77	*iso*-17:0	3.88
12:0 3OH	3.29	*iso*-12:0 3OH	3.28
*iso*-13:0 3OH	3.27	*iso*-13:0 3OH	3.08
*iso*-11:0	2.9	16:1 w9c	2.96
*iso*-17:0	2.49	14:0	2.9
*iso*-11:0 3OH	1.66	18:1 w9c	1.85
18:1 w9c	1.47	*iso*-11:0 3OH	1.82
*iso*-16:0	1.46	Unknown	1.53
Unknown	1.23	*iso*-16:0	1.51
14:0 ISO	1.06	18:1 w7c	1.13

**Figure 2:**
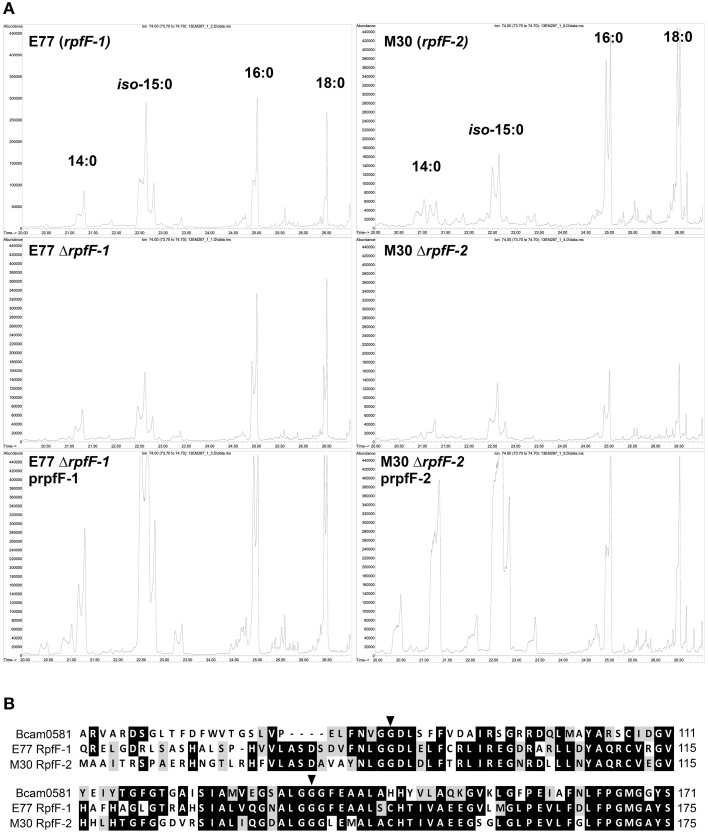
**(A)** Gas Chromatography analysis of FAs present in the supernatants of *S. maltophilia* E77 and M30, their respective Δ*rpfF* mutants and complemented strains. **(B)** Alignment of RpfF (partial sequence) in *B. cenocepacia* (Bcam0581) and *S. maltophilia* E77 and M30. Marked aminoacids indicate the residues responsible for thioesterase activity (Bi et al., 2012).

Extracellular FAs present in the supernatants of the two variant strains were also identified by GC/MS (Figure 2 and Figure S2). As for the cellular FAs, the FAs identified in the medium supernatants of the two strains were mostly the same, except for the signal DSF, which was not detected in M30 (*rpf*-2) supernatants (Huedo et al., 2014b). These included *iso*-15:0, 16:0, and 18:0. Deletion of *rpfF*-1 (strain E77) led to a decrease in *iso*-15:0 release, with levels restored (in excess) by *in trans* complementation (Figure 2A). The same effect was observed in the M30 strain, although in this case deletion of *rpfF*-2 led also to a significant decrease in the release of 16:0 and 18:0. These results suggest that, as described for the RpfF homolog Bcam0581 of *B. cenocepacia* (Bi et al., 2012), both *S. maltophilia* RpfF variants could have, apart from a dehydratase activity, an unspecific thioesterase activity that cleaves acyl-ACP bonds generating free FAs that are then released to the extracellular environment. Protein alignment (Figure 2B) shows that both RpfF variants conserve the amino-acids G87 and G139 responsible for the thioesterase activity in the *B. cenocepacia* ortholog (Bi et al., 2012). Overall, these results suggest that RpfF is not involved in the main synthetic pathway of cellular FAs (their composition not being affected by RpfF deletion), but participates in fatty-acid release and, therefore, determines to some extent the extracellular fatty-acid composition.

### Effect of the temperature, the medium and the presence of extracellular FAs on DSF production by *S. maltophilia*

Little is known about the growth conditions that modulate DSF production in *S. maltophilia*. In a recent report, we have shown that although only *rpf*-1 strains produce detectable levels of DSF under standard laboratory growth conditions, both synthase variants RpfF-1 and RpfF-2 are functional on DSF synthesis (Huedo et al., 2014b). Here, we aimed at investigating the conditions that may modulate such production, especially in *rpf*-2 strains. To this end, we developed a new, simple and efficient DSF-detection methodology based on a microtitter-plate bioassay (see Materials and Methods and Figure S1), which allowed us to accurately quantify DSF produced by *S. maltophilia* strains grown in different media and incubation temperatures. Using this method, we first quantified the DSF levels in culture supernatants from bacteria grown in LB medium at different temperatures (20, 25, 30, 37, and 42°C) for 48 h (Table 3). DSF production in strain E77 (*rpf*-1 variant) was temperature dependent and statistically significant only at 30°C (*p* < 0.05). Production was however detected also at 25 and 37°C, and not at 20 or 42°C. On the other hand, DSF was not detected in the supernatants of M30 (*rpf*-2 variant) grown in LB at any of the tested temperatures (Table 3).

**Table 3:** **Effect of temperature, medium composition and presence of extracellular fatty acids on DSF production by *S. maltophilia* strains E77 (*rpf*-1) and M30 (*rpf*-2)**.

**Experiment**	**Growth conditions**		**DSF production (μg/mL)**	
	**Medium**	**Temperature°C**	**E77**	**M30**
Temperature effect (LB)	LB	20	ND	ND
	LB	25	0.6±0.07	ND
	LB	30	1.4±0.2^*^	ND
	LB	37	0.25±0.03	ND
	LB	42	ND	ND
Medium composition effect (30°C)	M9	30	0.3±0.03	ND
	BM2	30	0.4±0.03	ND
	NYG	30	1.1±0.15	ND
	LB	30	1.4±0.2^*^	ND
Fatty acid supplementation effect (5 μM)	LB + 12:0	30	1.6±0.2	ND
	LB + 14:0	30	1.8±0.3	ND
	LB + *iso*-15:0	30	2.4±0.4^*^	ND
	LB + 16:0	30	1.35±0.1	ND
	LB + 18:0	30	ND	ND

*ND, Not detected*.

* *p < 0.05 (One-Way ANOVA and post-test)*.

The effect of medium composition at the optimal temperature (30°C) was then evaluated using a second rich medium (NYG) and two minimal media (BM2 and modified M9-salts). DSF production by E77 showed to be clearly higher in rich media (LB and NYG), while none of these conditions induced DSF production by the M30 strain (Table 3).

The finding that *S. maltophilia* RpfFs may have, just like *B. cenocepacia* Bcam0581 (Bi et al., 2012) and *Xcc* RpfF (Bi et al., 2014), a thiosterase activity that contributes to the accumulation of diverse FAs in the extracellular environment, led us to speculate that these FAs could contribute to modulate DSF production. To test this hypothesis, we supplemented E77 and M30 cultures with the FAs that were identified as predominant in their supernatants (12:0, 14:0, *iso*-15:0, 16:0, and 18:0, at 5 μM each). After incubation, DSF was quantified using the microtitter DSF bioassay. As negative control, the same volume of non-supplemented medium was in each case extracted and tested with the bioassay, resulting in no DSF activity (data not shown). Results showed that *iso*-15:0 significantly stimulates DSF production in E77 (Table 3). Interestingly, while 12:0, 14:0, and 16:0 have no significant effect on DSF synthesis in this strain, 18:0 appears to have an inhibitory effect. As expected, none of the FAs promoted DSF production in the M30 strain.

### *S. maltophilia rpf*-1 and *rpf*-2 variant strains cross talk producing DSF in a positive-feedback manner

**Figure 3:**
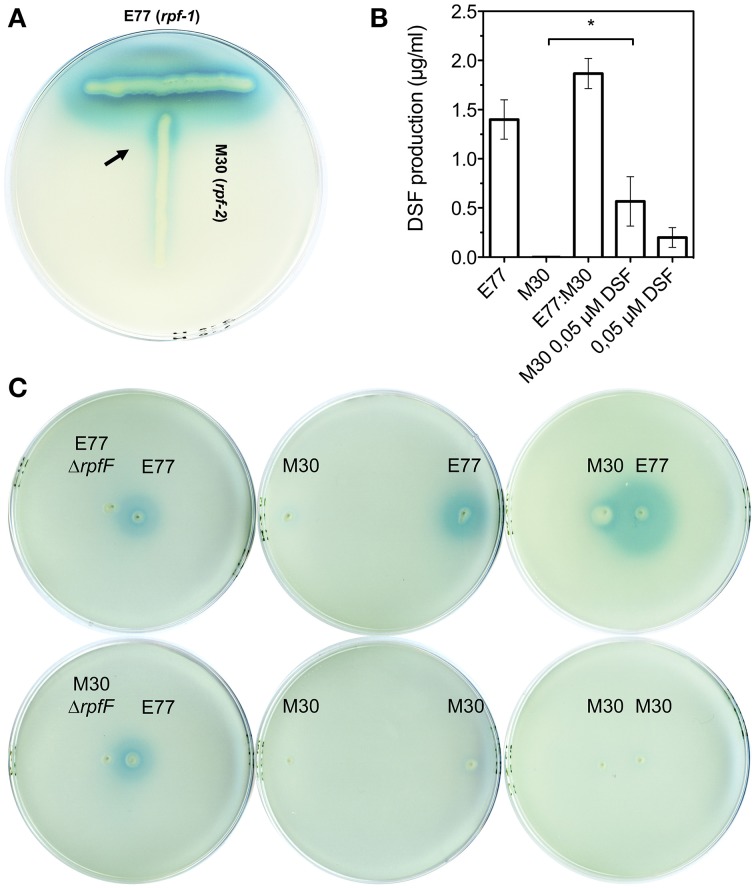
**(A)** T-seeded colony-based DSF bioassay of strains E77 and M30. Arrow indicates DSF production of M30 (*rpf*-2) strain upon detection of DSF molecules produced by E77 (*rpf*-1). **(B)** DSF quantification of supernatants from axenic cultures of E77 and M30, a mixed culture E77:M30 (1:1), an axenic culture of M30 supplemented with synthetic DSF at 0.05 μM final concentration and a corresponding control (equal volume of LB broth containing 0.05 μM DSF), using the microtitter DSF bioassay; ^*^p < 0.05 by One-Way ANOVA and post-test. **(C)** Colony-based DSF bioassay of E77, M30 and their respective Δ*rpfF* mutants seeded at different distances on the same agar plate.

Since we had previously observed that the RpfF-2 variant is functional when the RpfF-2:RpfC-2 stoichiometric ratio is favorable to the RpfF-2 protein (RpfF-2 > RpfC-2) (Huedo et al., 2014b), we now aimed to determine the conditions under which the RpfF-2/RpfC-2 complex could dissociate and synthesize DSF in a wild-type background. We have observed *in vitro* that, when colonies of *S. maltophilia* E77 and M30 are grown at a distance at which the halo of DSF production from E77 invades the growing zone of M30, the latter begins to produce DSF (Figure 3). Additionally, it seems that this synergism is reciprocal, since both strains produce higher levels of DSF when are seeded closely than when are seeded separately (Figures 3A,C). Also, when E77 is grown close to a Δ*rpfF*-1 or Δ*rpfF*-2 mutant colony, no increment in DSF production is observed (Figure 3C), which corroborates that under wild-type conditions the observed synergism in DSF production depends only on the DSF molecule (Figure 3C). Furthermore, mixed cultures of E77 and M30 grown in LB broth at 30°C for 48 h displayed higher DSF production than E77 axenic cultures (Figure 3B). Finally, supplementation of M30 cultures in LB with 0.05 μM DSF significantly triggers DSF production (*p* < 0.05) after 48 h of growth (Figure 3B). Together, these results confirm that RpfC-2 is able to sense DSF, that DSF is produced in a positive feedback-manner and that the variant groups *rpf*-1 and *rpf*-2 produce DSF synergistically if they grow in close contact.

### *S. maltophilia rpf*-1 and *rpf*-2 variant strains act synergistically to promote virulence in a zebrafish infection model

The observation that the two populations produced DSF synergistically led us to investigate whether these strains would also cooperate to develop virulence *in vivo*. To this end, we performed infection experiments in a recently developed adult zebrafish model using groups of 16 fishes. Each group was inoculated with either: an axenic inoculum of E77, an axenic inoculum of M30, a mixed inoculum of E77 and M30 wild type (1:1) and a mixed inoculum of Δ*rpfF* mutant strains Δ*rpfF*-1:Δ*rpfF*-2 (1:1), all of them with the same total bacterial load (~5 × 10^8^ CFU). Control animals were injected with PBS.

**Figure 4:**
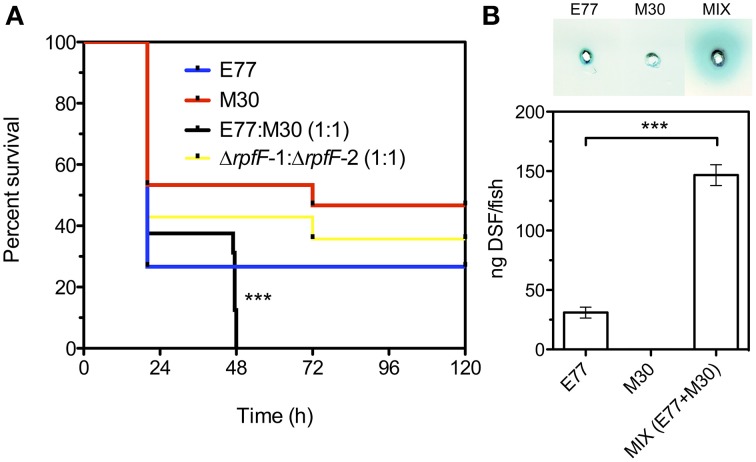
**(A)** Virulence determination of E77, M30, a mixed inoculum of E77 and M30 (1:1) and a mixed inoculum of E77 Δ*rpfF-1* and M30 Δ*rpfF-2* (1:1), in the adult zebrafish model; ^***^p < 0.001 (log-rank test). **(B)** Supernatant-based DSF bioassay of tissue extracted from sacrificed fishes 48 h after infection with E77, M30 and a mixed inoculum (E77:M30, 1:1) (top) and quantification of DSF in the samples using the microtitter bioassay (bottom); ^***^p < 0.001 by One-Way ANOVA and post-test.

The infection experiments showed that when animals were infected with the mixed wild-type inoculum all fishes (*n* = 16) died within the first 48 h, whereas the fishes infected with the axenic cultures E77 and M30 showed a survival between 20 and 50% at 120 hpi (Figure 4A). Moreover, when fishes were infected with a mixed inoculum consisting of E77 Δ*rpfF*-1 and M30 Δ*rpfF*-2 the survival ratio was close to 40% at 120 hpi, demonstrating that RpfF is an essential trait for the virulence capacity of the *S. maltophilia* population (Figure 4A). Symptoms of disease as well as fish behavior were evaluated during the days of infection. Interestingly, fishes infected with the mixed wild-type inoculum showed a drastic change in behavior (spasms, compromised swimming and sudden death), especially between 24 and 48 h (data not shown).

To assess DSF production during the *in vivo* infection and investigate the eventual correlation with the lethal capacity of the inocula, two fishes from each condition were sacrificed at 48 hpi and the DSF inside the infected animals was quantified. The condition that leads to 100% mortality (mixed inoculum of E77 and M30 wt) showed significant increment (*p* < 0.001) in DSF content relative to the axenic inoculum with E77 (Figure 4B), showing a clear correlation between *in vivo* DSF production and virulence capacity. No DSF was detected in PBS-injected animals.

## Discussion

Although the DSF-QS system has been deeply studied in Xanthomonadales species (Deng et al., 2011), the biosynthetic pathway of DSF, its regulation and the way in which the sensor RpfC perceives DSF signals remain unclear. This is especially true for the opportunistic human pathogen *S. maltophilia*, in which DSF-QS regulation appears to be particularly complex. In a recent work, our group demonstrated that, in contrast to other species featuring the DSF-QS system, in *S. maltophilia* there exist two populations—designated *rpf*-1 and *rpf*-2—that differentially regulate DSF production and detection (Huedo et al., 2014b). In *rpf*-1 variant strains DSF-QS regulation seems to be reasonably similar to that described in *Xcc*. Phylogenetic analysis of the *rpf* cluster among Xanthomonadales further confirms the similarity between the *S. maltophilia rpf*-1 cluster variant and the *Xcc rpf* cluster, both in terms of sequence identity and genomic organization (Figure 1). On the other hand, the *rpf*-2 cluster organization is more similar to that of the *rpf* clusters of *Pseudoxanthomonas* spp., *Arenimonas* spp. and *Lysobacter* spp. (Figure 1), bacteria commonly isolated from the rhizosphere. The high competition and enhanced horizontal gene transfer rates characteristic of this competitive ecological niche (Berg et al., 2005) might have contributed to the genetic and metabolic variability observed in *S. maltophilia*, also reflected in DSF-QS regulation. The aim of this study was to get further insight into the complexity of the DSF-QS system of *S. maltophilia*.

The elevated proportion of *iso*-15:0 found in the analysis of total cellular FAs indicates that the identified methyl ester derives from membrane phospholipids. In line with this result, *iso*-15:0 has been also reported as the most abundant FA in several *Xanthomonas* species, including *Xcc, Xanthomonas oryzae* pv. *oryzae* (*Xoo*) and *X. axonopodis* pv. *citri* (*Xac*), among others (Vauterin et al., 1996). Surprisingly, *iso*-15:0 has been also found in similar proportion in total phospholipids of Gram-positive bacteria such as *Bacillus* spp. PS3 (34%) (Kagawa et al., 1978) and is actually considered a biomarker phospholipid FA for the Gram-positive group (Kaur et al., 2005). In this context, it is remarkable that among the Gram-negative group *iso*-15:0 appears to be present only, and with similar relative abundances, in DSF-producer bacteria such as *S. maltophilia* (Table 2) and *Xanthomonas* spp. (Vauterin et al., 1996; Bi et al., 2014). By similarity to protein Bcam0581 from *B. cenocepacia* (which produces *cis*-2-dodecenoic acid or BDSF), RpfF has been postulated to have a double acyl-ACP dehydratase and thioesterase activity, which would catalyze the conversion of (*R*)-3-hydroxy-11-methyl-dodecanoyl-ACP to DSF in two steps (Bi et al., 2012). Furthermore, the thioesterase activity of both Bcam0581 and the RpfF from *Xcc* has been observed to be unspecific, cleaving a variety of acyl-ACP substrates and thus generating free medium- and long-length FAs that are then released to the medium (Bi et al., 2012, 2014). Our analysis of extracellular FAs suggests that the two RpfF variants of *S. maltophilia* do also have a thioesterase activity that is not specific for the DSF precursor but may also cleave, at least, the acyl-ACP form of *iso*-15:0 (Figure 2A). Interestingly, the putative DSF precursor (*R*)-3-hydroxy-11-methyl-dodecanoyl-ACP is also a precursor of *iso*-15:0 in the fatty-acid synthesis cycle (Heath et al., 2002) (note that methylation of *iso*-branched FAs originates from the primer for biosynthesis, which derives from valine in odd-numbered chains and leucine in even-numbered chains). The potential connection between DSF and *iso*-15:0 biosynthesis leads us to speculate on a corresponding potential connection between DSF production and membrane synthesis.

**Figure 5:**
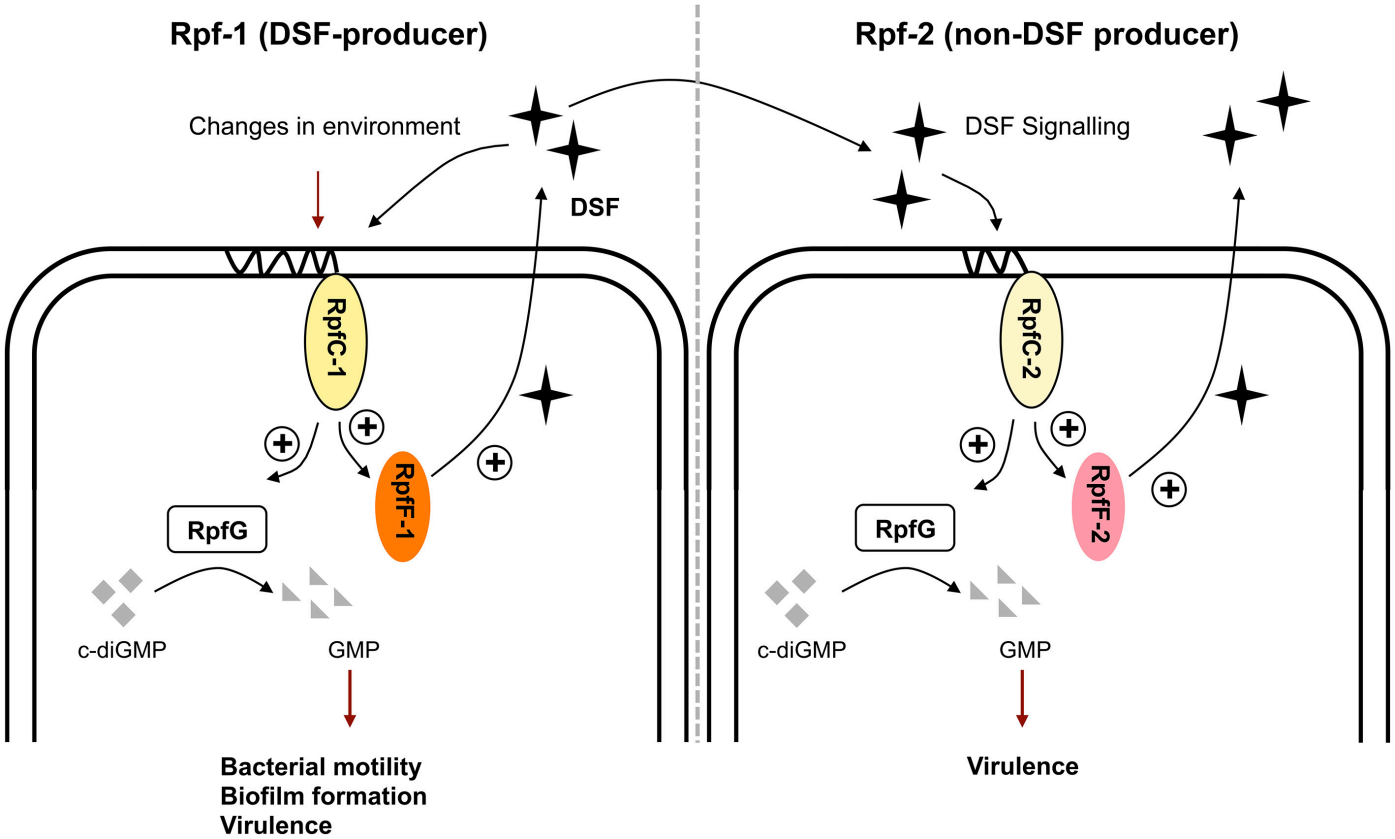
**Schematic model of the DSF-QS proposed for *S. maltophilia rpf*-1 (DSF-producer) and *rpf*-2 (non-DSF producer) variant strains**. Rpf-1: RpfC-1 (containing 10 TMR) allows basal activation of RpfF-1, with subsequent DSF production, when reaching high cellular density. Changes in the environment including temperature, nutrients and presence of extracellular fatty acids modulate DSF production. The DSF-QS system controls bacterial motility, biofilm dispersion, and virulence. Rpf-2: RpfC-2 (containing 5 TMR) does not allow basal DSF production. None of the mentioned factors stimulate DSF production in these variant strains. Activation of RpfF-2 and subsequent DSF production happens only upon detection of DSF itself, likely coming from neighbor DSF-producer strains (e.g., *Xcc* or *S. maltophlia rpf*-1). In this situation, *rpf*-2 strains regulate synergistically virulence capacity and DSF production in co-infection with *rpf*-1 variant strains.

In this context, we have observed that, besides temperature and medium composition, the presence of RpfF-dependent FAs in the medium can modulate DSF production in variant 1 (Table 3). Indeed, it seems that the presence of exogenous *iso*-15:0 has a stimulatory effect on DSF synthesis in this variant strain. Since RpfC has both a sensor and a RpfF-repression function (Cheng et al., 2010; Huedo et al., 2014b), we hypothesize that RpfC-1 may be able to detect *iso*-15:0—and possibly other FAs—leading to the liberation of RpfF-1 and subsequent synthesis of DSF. On the contrary, RpfC-2—with a shorter sensor input domain containing only five TMRs (Huedo et al., 2014b)—would be incompetent for promiscuous perception, resulting in a permanent repression of the RpfF-2 synthase despite the presence of *iso*-15:0 and other FAs in the medium. Indeed, it appears that the RpfF-2/RpfC-2 complex is able to dissociate, and thus produce DSF, only upon detection of DSF-itself (Figure 3), indicating that, contrary to *rpf*-1, the sensor complex in *rpf*-2 is more specific. Recent findings on *X. fastidiosa* (RpfC-2-like variant with 5 TMR) show that in this species RpfF is required for DSF detection (Ionescu et al., 2013). It seems thus clear that some initial DSF production must therefore occur in order to trigger DSF synthesis by *rpf*-2 strains. The specific conditions that would facilitate this initial DSF production in axenic cultures have not yet been elucidated. However, it is well established that in the natural environments of *S. maltophilia* there exists extensive intra- and inter-population competence and communication (Wang et al., 2004), even between organisms from different domains (Boon et al., 2008). Since bacterial species sharing DSF-QS are almost ubiquitous and frequently share ecological niches, it is likely that *rpf*-2 variant strains will often be in contact with DSF-producer bacteria (e.g., *Xcc* or *S*. *maltophilia rpf*-1 variant). In this situation, a DSF-producer strain would act as a starter strain, triggering the reciprocal DSF-communication by synthesizing the initial DSF molecules (Figure 5). Interestingly, we have observed that, besides showing *in vitro* synergism in DSF production (Figure 3), the two variant-groups communicate enhancing the virulence capacity of a mixed *S. maltophilia* population during *in vivo* infection (Figure 4). It seems that *rpf*-2 strains have evolved as a receptor group in this DSF communication, showing a lethargic DSF-deficient phenotype in axenic conditions that would save energy and allow a finer regulation of processes related to DSF communication. Recent studies have evidenced that within bacterial communities there exist individual cells that turn off their QS communication—by accumulation of diverse mutations—and take advantage of public goods, thus saving energy. This recently discovered behavior is termed “social cheating” and has raised much interest. It has been observed that several *P. aeruginosa* isolates from cystic fibrosis patients accumulate mutations in the gene encoding for the QS regulator LasR (Smith et al., 2006; Tingpej et al., 2007; Hoffman et al., 2009). Although *lasR* mutants are not able to trigger the QS response (Haas, 2006), they frequently coexist with wild-type isolates and take advantages from their intact QS-regulation (Sandoz et al., 2007). Inspired by this phenomenon, we speculate that *S. maltophilia rpf*-2 variant strains may constitute a conserved, therefore successful, population of “social cheaters.” The specific advantages (besides energy saving) and disadvantages (in the absence of DSF-producer bacteria) of this behavior are yet to be elucidated.

Interspecies communication mediated by DSF-like signal molecules appears to be common in Proteobacteria, not only among Xanthomonadales (Deng et al., 2011). For example, it has been shown that DSF produced by *S. maltophilia* modulates the behavior of *P. aeruginosa*, including biofilm formation and antibiotic resistance (Ryan et al., 2008) and virulence and persistence in lungs of CF patients (Twomey et al., 2012). *Xcc* can also regulate certain virulence factors in response to BDSF produced by *B. cenocepacia* (Deng et al., 2010). It has been also reported that the DSF-related signal *cis*-DA (*cis*-decenoic acid) produced by *P. aeruginosa* (Davies and Marques, 2009; Amari et al., 2013) induces biofilm dispersion of several Gram-negative and Gram-positive bacteria (Davies and Marques, 2009). Recently, our group has reported that *S. maltophilia* can also respond to acyl homoserine lactone (AHL) signals produced by *P. aeruginosa* and regulate swarming motility (Martínez et al., 2015). Additionally, the morphological transition of *C. albicans* is inhibited in the presence of various DSF-like molecules (Wang et al., 2004; Boon et al., 2008; Deng et al., 2010) including SDSF (*trans*-2-decenoic acid), a FA produced by *Streptococcus mutans* (Vílchez et al., 2010). Clearly, FA-mediated communication is largely distributed in bacteria and has a very relevant interspecies component. Combining these data with our results, it becomes obvious that complex population processes, such as QS, are not well addressed when they are studied only in axenic conditions.

### Conflict of interest statement

The authors declare that the research was conducted in the absence of any commercial or financial relationships that could be construed as a potential conflict of interest.

## Supplementary material

File S1 **Detection of putative recombination breakpoints in the *rpf* cluster**.

Figure S1 **DSF microtitter bioassay**. **(Top)** Wells containing DSF-reporter solution (see Materials and Methods) inoculated with increasing concentrations (0.05, 0.1, 0.2, 0.4, 0.8, and 1.6 mM) of synthetic DSF (Sigma). **(Bottom)** Calibration curve (*rI* = 0.9787) generated by reading the absorbance of the wells at 620 nm after incubation at 28°C for 24 h.

Figure S2 **Mass spectra of the Gas Chromatography peaks corresponding to the fatty acids 14:0 (21.2 min), *iso*-15:0 (22.5 min), 16:0 (25.0 min), and 18:0 (26.5 min)**.

Table S1 **Total cellular fatty acids in *S. maltophilia* mutants E77 Δ*rpfF*-1 and M30 Δ*rpfF*-2**.
