# Supplementary material for: Decoding the genetic and functional diversity of the DSF quorum-sensing system in Stenotrophomonas maltophilia
Source: Front Microbiol. 2015 Jul 28;6:761. doi: 10.3389/fmicb.2015.00761 (PMC4517397; doi:10.3389/fmicb.2015.00761)
Supplement: File S1 — Detection of putative recombination breakpoints in the rpf cluster. [file DataSheet1.DOCX]

**Detection of putative recombination breakpoints in the *rpf* cluster**

**Sequences.** The genetic sequence encoding for the *rpf* cluster were extracted from the genomes of the Xanthomonadales strains listed in Data Sheet Table S1 downloaded from the NCBI Web site (<http://www.ncbi.nlm.nih.gov>).

**Data Sheet Table S1**. Genomic sequence fragments used in the analysis.

| Specie and strain | Genbank accession to genome | *rpf* cluster coordinates | *rpf* cluster-type organization* |
| --- | --- | --- | --- |
| Genomes with complete information | | | |
| *Stenotrophomonas maltophilia* K279a | NC_010943.1 | 2265201-2271824 | K279a (*rpf*-1) |
| *Stenotrophomonas rhizophila* DSM14405 | CP007597.1 | 2467670-2475799 |  |
| *Xanthomonas campestris pv. campestris* ATCC 33913 | AE008922.1 | 2158339-2165029 | *Xcc* |
| *Xanthomonas oryzae pv. oryzae* MAFF311018 | AP008229.1 | 3070367-3076788 | M30 |
| *Xanthomonas oryzae pv. oryzicola* BLS256 | CP003057.1 | 2256509-2262938 | M30 |
| *Xanthomonas citri subsp. citri* Aw12879 | CP003778.1 | 2823953-2830059 | M30 |
| *Xanthomonas axonopodis* Xac29-1 | CP004399.1 | 2180325-2186430 | M30 |
| *Xylella fastidiosa* 9a5c | AE003849.1 | 298829-300496  1068636-1072606 |  |
| *Pseudoxanthomonas spadix* BD-a59 | CP003093.2 | 1867889-1874350 | M30 |
| *Pseudoxanthomonas suwonensis* 11-1 | CP002446.1 | 1873106-1879284 | M30 |
| *Xanthomonas albilineans* GPE_PC73 | FP565176.1 | 1555939-1562002 | M30 |
| *Xanthomonas fuscans subsp. fuscans* 4834-R | FO681494.1 | 2312557-2319255 | *Xcc* |
| Genomes in contigs (only the contig containing *rpf* cluster is indicated) | | | |
| *Stenotrophomonas maltophilia* M30 | JELS02000111.1 | 71043-77143 | M30 (*rpf*-2) |
| *Xanthomonas arboricola pv. pruni* MAFF 301420 | BAVC01000071.1 | 19094-25495 | M30 |
| *Lysobacter dokdonensis* DS-58 | JRKJ01000010.1 | 85899-92019 | M30 |
| *Xanthomonas hortorum pv. carotae* M081 | AEEU01000119.1  AEEU01000120.1 | 6862-11094  4875-6557 | M30 |
| *Xanthomonas translucens pv. graminis* ART-Xtg29 | ANGG01000513.1 | 3017-9061 | M30 |
| *Arenimonas malthae* CC-JY-1 | AVCH01000104.1 | 62871-68992 | M30 |
| *Xanthomonas axonopodis pv. phaseoli* NCPPB 381 | JTKK01000096.1 | 6151-12849 | *Xcc* |

**rpf* cluster type based on gene organization similarities with model *S. maltophilia* strains K279a or M30 or *X. campestris pv. campestris* ATCC 33913 (*Xcc*).

**Sequence alignments.** The alignment of the entire *rpf* operon was reconstructed using program MEGA6 (Tamura et al., 2013). Due to differences in operon organization, sequences from *S. rhizophila* and *X. fastidiosa* were not included in this analysis. Coding sequences were aligned separately using MEGA6 for intragenic recombination analysis. For coding sequences, the amino-acidic sequences were aligned and then the resulting alignments were back translated into multiple alignments of nucleotide sequences using MEGA6.

**Detection of recombination.** A model-based approach, the Genetic Algorithm for Recombination Detection (GARD) (Kosakovsky Pond et al., 2006), was used to search for putative breakpoints delimiting sequence regions having distinct phylogenies. This analysis compares the goodness of fit of phylogenies inferred from alignment fragments under the maximum likelihood framework using the corrected Akaike Information Criterion (AIC). To further validate breakpoints detected by GARD, the Kishino-Hasegawa (KH) test was applied to verify whether adjacent sequence fragments yield statistically different tree topologies. GARD program is freely available at <http://www.datamonkey.org/GARD/>, and it includes the KH test for tree congruence.

We detected some evidence of recombination events within the four genes in the *rpf* cluster. The GARD algorithm and KH test identified multiple recombination breakpoints in the *rpfB*, *rpfC* and *rpfG* genes. In contrast, no recombination was detected within the *rpfF* gene when a single gene alignment was used. However, when the full operon sequence were used in a single alignment, GARD analysis predicted two recombination break point at both the 5' and 3' ends of the *rpfF* gene.

**Data Sheet Figure S1.** Schematic representation of the main *rpf* operon variants in Xanthomonadales where the vertical dashed lines represent predicted recombination breakpoint sites using the GARD program. Table (right) shows the results of the GARD tests for recombination applied to each gene or the full operon. Only those sites with significant *P*-value in the KH test are represented in the figure and indentified with circled numbers both in the table and figure (***<0.01; **<0.05; NS not significant). ΔAIC_c_: improvement in AIC_c_ of the breakpoint-partitioned model over a no-recombination single phylogeny model.

References

Kosakovsky Pond, S. L., Posada, D., Gravenor, M. B., Woelk, C. H., and Frost, S. D. W. (2006). GARD: a genetic algorithm for recombination detection. *Bioinforma. Oxf. Engl.* 22, 3096–3098. doi:10.1093/bioinformatics/btl474.

Tamura, K., Stecher, G., Peterson, D., Filipski, A., and Kumar, S. (2013). MEGA6: Molecular Evolutionary Genetics Analysis version 6.0. *Mol. Biol. Evol.* 30, 2725–2729. doi:10.1093/molbev/mst197.
