# Supplementary figures and images for: Decoding the genetic and functional diversity of the DSF quorum-sensing system in Stenotrophomonas maltophilia
Source: Front Microbiol. 2015 Jul 28;6:761. doi: 10.3389/fmicb.2015.00761 (PMC4517397; doi:10.3389/fmicb.2015.00761)

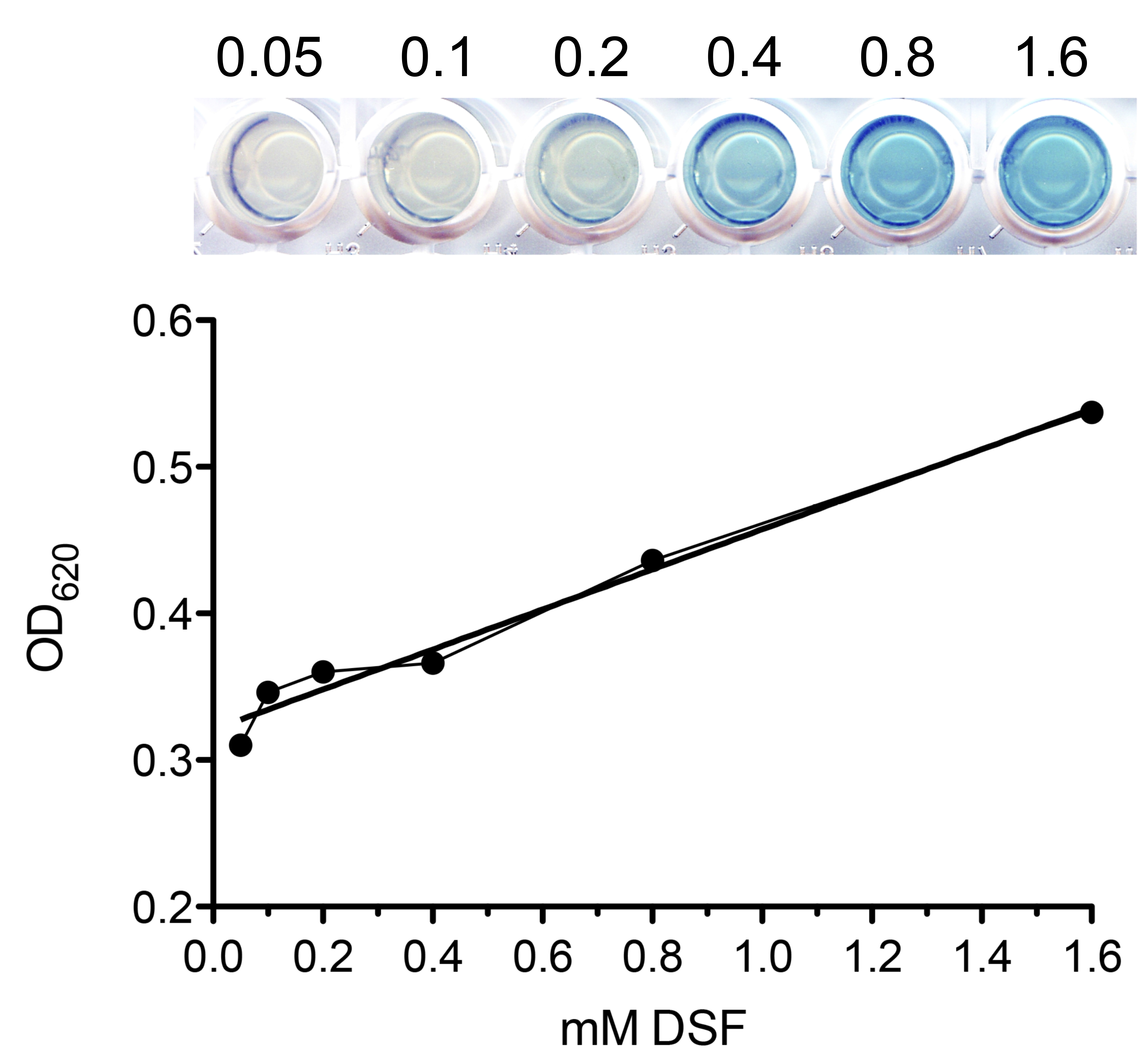

Supplement: Figure S1 — DSF microtitter bioassay. (Top) Wells containing DSF-reporter solution (see Materials and Methods) inoculated with increasing concentrations (0.05, 0.1, 0.2, 0.4, 0.8, and 1.6 mM) of synthetic DSF (Sigma). (Bottom) Calibration curve (rI = 0.9787) generated by reading the absorbance of the wells at 620 nm after incubation at 28°C for 24 h. [file Image1.JPEG]

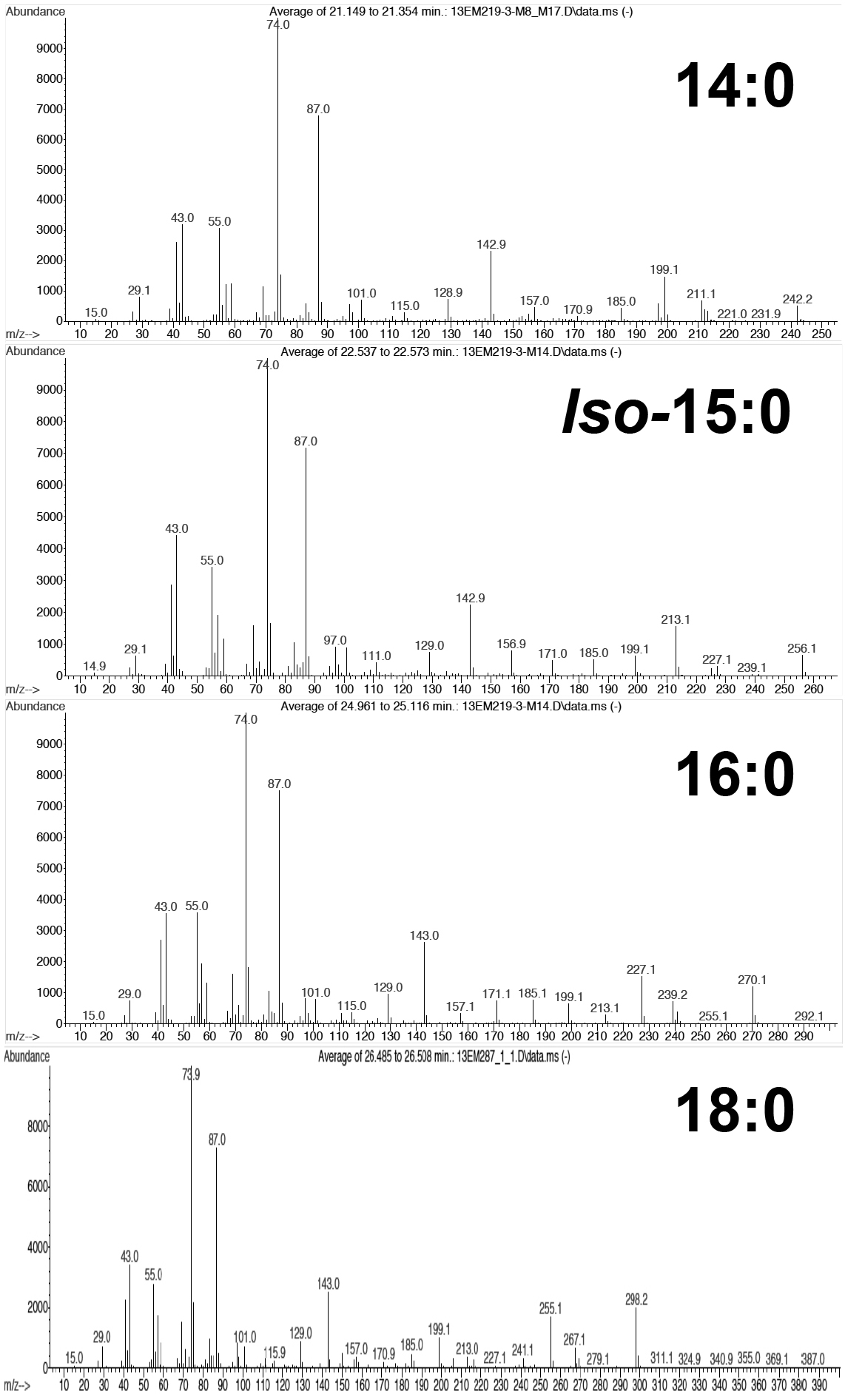

Supplement: Figure S2 — Mass spectra of the Gas Chromatography peaks corresponding to the fatty acids 14:0 (21.2 min), iso-15:0 (22.5 min), 16:0 (25.0 min), and 18:0 (26.5 min). [file Image2.JPEG]
