# Supplementary material for: Decoding the genetic and functional diversity of the DSF quorum-sensing system in Stenotrophomonas maltophilia
Source: Front Microbiol. 2015 Jul 28;6:761. doi: 10.3389/fmicb.2015.00761 (PMC4517397; doi:10.3389/fmicb.2015.00761)
Supplement: Table S1 — Total cellular fatty acids in S. maltophilia mutants E77 ΔrpfF-1 and M30 ΔrpfF-2. [file Table1.DOCX]

**Table S1.** Total cellular fatty acids in *S. maltophilia* mutants E77 ∆*rpf-*1 and M30 ∆*rpf-*2.

| E77 ∆*rpf*-1 | | M30 ∆*rpf-*2 | |
| --- | --- | --- | --- |
| Fatty acid | Percentage | Fatty acid | Percentage |
| *iso-*15:0 | 32.55 | *iso-*15:0 | 32.95 |
| *anteiso-*15:0 | 12.66 | *iso-*15:0 2OH o 16:1 w7c | 11.98 |
| *iso-*15:0 2OH o 16:1 w7c | 12.45 | *anteiso-*15:0 | 10.25 |
| 16:0 | 6.05 | 16:0 | 9.12 |
| *iso*-17:1 w9c | 3.99 | *iso-*11:0 | 4.23 |
| 14:0 | 3.77 | *iso*-17:0 | 3.85 |
| 16:1 w9c | 3.51 | *iso*-17:1 w9c | 3.68 |
| *iso-*13:0 3OH | 3.42 | *iso*-12:0 3OH | 3.24 |
| 12:0 3OH | 3.34 | *iso-*13:0 3OH | 2.98 |
| *iso*-17:0 | 2.56 | 16:1 w9c | 2.96 |
| *iso-*11:0 | 1.72 | 14:0 | 2.62 |
| *iso-*11:0 3OH | 1.66 | *iso-*11:0 3OH | 1.88 |
| *iso-*16:0 | 1.55 | 18:1 w9c | 1.62 |
| 18:1 w9c | 1.39 | *iso-*16:0 | 1.51 |
| Unknown | 1.23 | Unknown | 1.38 |
| 14:0 ISO | 1.10 | 18:1 w7c | 1.25 |
